# Short-term effects of pimobendan in dogs with preclinical myxomatous mitral valve disease

**DOI:** 10.3389/fvets.2026.1903502

**Published:** 2026-08-18

**Authors:** Irene Apolloni, Stefano Oricco, Serena Crosara, Giorgia Cavalleri, Marco Poggi, Cecilia Quintavalla

**Affiliations:** 1 Department of Veterinary Science, University of Parma, Parma, Italy; 2 Centro Veterinario Imperiese, Imperia, Italy

**Keywords:** canine, echocardiography, inotropic, MMVD, remodeling

## Abstract

**Introduction:**

Pimobendan has been shown to delay the onset of congestive heart failure and prolong survival in dogs affected by myxomatous mitral valve disease (MMVD). However, little information is available regarding its short-term echocardiographic effects in dogs with preclinical disease. The aim of this prospective multicenter observational study was to evaluate the effect of pimobendan on selected echocardiographic indices after 7 days of therapy in dogs with American College of Veterinary Internal Medicine stage B2 MMVD.

**Methods:**

Forty-two client-owned dogs received pimobendan orally at 0.25–0.3 mg/kg every 12 h and underwent echocardiographic examination before treatment initiation and after 7 days.

**Results:**

Significant reduction was observed in several indices of left atrial and left ventricular remodeling, including normalized left atrial diameter (*p* < 0.001), left atrial-to-aortic ratio (*p* < 0.001), maximal and minimal left atrial volumes (*p* < 0.001 and *p* = 0.008, respectively), left atrial stroke volume (*p* = 0.011), and normalized left ventricular internal diameters in diastole and systole (both *p* < 0.001). Significant decrease was also observed in fractional shortening (*p* = 0.007), peak velocity of early diastolic transmitral flow (E) (*p* = 0.001), E velocity-to-early diastolic myocardial peak velocity ratio (*p* = 0.031), mitral-to-aortic velocity time integral ratio (*p* = 0.045), and left ventricular early inflow-outflow index (*p* < 0.001), whereas pulmonic velocity-time integral (*p* < 0.001) and systolic myocardial peak velocity (*p* < 0.001) significantly increased. No significant differences were detected for regurgitant volume, regurgitant fraction, ejection fraction, myocardial performance index, isovolumic relaxation time, E-to-isovolumic relaxation time ratio, E deceleration, or aortic velocity-time integral. At the 1-week re-evaluation, 22/42 dogs (52.4%) showed reverse remodeling, with echocardiographic indices returning to values within the range consistent with ACVIM stage B1.

**Discussion:**

These findings suggest that pimobendan administration is associated with early improvement in echocardiographic indices of left heart remodeling in dogs with ACVIM stage B2 MMVD and may reduce regurgitant volume shortly after treatment initiation.

## Introduction

Pimobendan is a calcium sensitizer with positive inotropic and vasodilatory properties. Pivotal clinical trials have demonstrated that it delays the onset of congestive heart failure (CHF) in preclinical dogs with myxomatous mitral valve disease (MMVD) and prolongs survival in dogs with MMVD and CHF (1–3). These findings have markedly changed the therapeutic management of MMVD and support its widespread clinical use in dogs (4). In addition to its effects on clinical outcome, pimobendan has been associated with echocardiographic changes consistent with cardiac reverse remodeling in dogs with preclinical MMVD. Previous studies have reported reductions in left atrial (LA) and left ventricular (LV) dimensions following treatment, along with improvements in systolic function indices (5, 6).

Moreover, a decrease in mitral regurgitation severity has been observed after 7 days of treatment (7), alongside early improvement in echocardiographic measures of systolic performance, such as end-systolic volume and ejection fraction (8).

Overall, limited studies have specifically investigated the short-term effects of pimobendan therapy in dogs with MMVD at the American College of Veterinary Internal Medicine (ACVIM) stage B2. Therefore, the aim of this study was to evaluate the short-term effects of pimobendan, administered orally at 0.25–0.3 mg/kg every 12 h, on left atrial and ventricular size and function in dogs with preclinical MMVD.

We hypothesized that pimobendan administration would result in measurable improvements in echocardiographic indices of left ventricular chamber dimensions, mitral regurgitation severity, flow patterns, and systolic and diastolic function.

## Materials and methods

### Study design and population

This was a multicenter, prospective short-term study evaluating the effects of treatment in client-owned dogs diagnosed with ACVIM stage B2 MMVD. Dogs were recruited from the Cardiology Unit of the Veterinary Teaching Hospital of the University of Parma and from the Centro Veterinario Imperiese, between January 2022 and December 2025.

At the time of diagnosis, owners were informed about the study protocol and provided written consent for their dogs’ participation, including a scheduled re-evaluation after 1 week of treatment with pimobendan. Ethical approval was granted by the Institutional Animal Ethics Committee (protocol number 11/CESA/2024).

Dogs were included if they fulfilled the following criteria: diagnosis of ACVIM stage B2 MMVD at the time of presentation, defined as the presence of the typical thickening and prolapse of the mitral valve and left cardiac remodeling, according to the ACVIM guidelines (4). The dogs had to be free of prior administration of cardiac medications; free of known concurrent systemic diseases that could affect cardiovascular function; and free of clinically relevant arrhythmias. Moreover, the owners had to be available to return to the hospital for a recheck after 1 week. Dogs not meeting all inclusion criteria were excluded from the study.

All dogs had a second echocardiographic examination after receiving pimobendan 0.25–0.3 mg/kg PO q12h for 7 days.

### Data collection

At the time of stage B2 diagnosis, the following data were collected: breed, age, body weight, sex, and dosage of pimobendan.

Echocardiographic examinations were performed by expert operators at the diagnosis and 7 days after the beginning of pimobendan. All echocardiographic measurements were performed by a single operator, and intra-observer variability was assessed.

The exams were performed on unsedated dogs, gently restrained in both right and left lateral recumbency. Standard 2-dimensional, M-mode, and Doppler echocardiographic images were acquired. All measurements were taken over 3 cardiac cycles, and the median was used for statistical purpose.

From the right parasternal 4-chamber view, the left atrial diameter was measured parallel to the mitral annular plane, at the middle part of the LA, and was then normalized on the body weight (LADn) (9). From the 5-chamber view, the aortic annulus diameter was measured at the insertion of the leaflet in mid-systole. The normalized left ventricular internal diameters in diastole (LVIDDn) and systole (LVDSn) were obtained using M-mode imaging, and normalization on body weight was calculated with the reported formula (10). The fractional shortening (FS%) was calculated from the M-mode LV diameters. The left atrial to aortic root ratio (LA/Ao) was measured from the right parasternal short-axis view at the heart base (11). From the left parasternal apical 4-chamber view, the LA volumes were measured using the area-length method at two different points of the cardiac cycle: just before the mitral valve opening (maximal LA volume; LAV_max_), and at the mitral valve closure (minimal LA volume; LAV_min_) (12); the left atrial stroke volume (LA-SV) was calculated as the difference between LAV_max_ and LAV_min_. Peak velocity of early diastolic transmitral flow (E_vel_) and its deceleration (E_dec_) were obtained by pulsed-wave Doppler with the sample volume at the tips of the open mitral leaflets. The velocity–time integral of the mitral inflow (VTI_Mit_) was measured by tracing the Doppler spectral envelope. Tissue Doppler was obtained from the LV free wall, and the peak velocities of early diastolic (E′), and systolic (S′) myocardial velocity were measured. The E to E′ ratio (E/E′) was calculated. From the tissue Doppler signal, the isovolumic relaxation time (IVRT) was measured, and the Myocardial performance index (MPI) was calculated with the following formula: (isovolumic contraction time + IVRT)/ejection time. The E-to-IVRT ratio (E/IVRT) was also calculated.

Pulmonic flow was obtained with pulsed-wave Doppler from the right parasternal short-axis view, placing the sample volume on the annular plane; the aortic flow was recorded with pulsed-wave Doppler from the subcostal view, with the sample volume positioned on the annular plane. The velocity-time integral of pulmonic (VTI_PA_) and aortic (VTI_Ao_) flows was recorded.

The LV volumes were obtained with the Simpson’s method from the right parasternal 4-chamber view, and the ejection fraction (EF%) was calculated. The regurgitant volume (RV) was calculated as: (end-diastolic LV volume - end-systolic LV volume)-(aortic anulus area x VTI_Ao_). The regurgitant fraction (RF) was calculated with the following formula: [(end-diastolic LV volume-end-systolic LV volume)-(aortic anulus area x VTI_Ao_)]/[(end-diastolic LV volume-end-systolic LV volume)].

Two Doppler indices of mitral regurgitation severity were tested: the mitral to aortic flow velocity time integral ratio (MAVIR = VTI_Mit_ /VTI_Ao_) (13), and the left ventricular early inflow-outflow index (LVEIO = E_Vel_/VTI_Ao_) (14).

### Statistical analysis

Statistical analysis was performed using R^c^ (version 4.6.0, The R Foundation for Statistical Computing; RStudio, version 2026.04.0 + 526, Posit Software, PBC).

**Figure 1:**
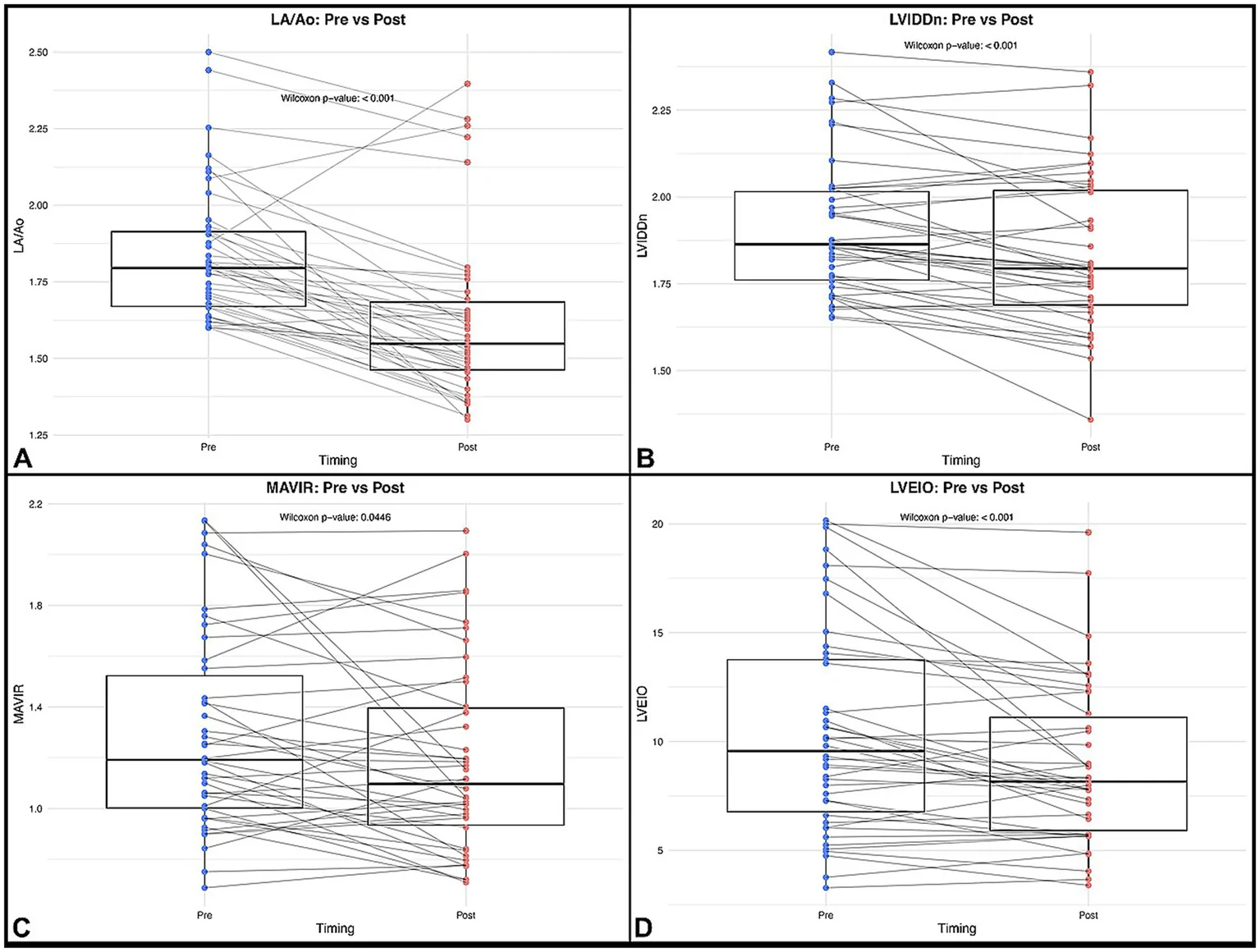
Boxplots of LA/Ao **(A)**, LVIDDn **(B)**, MAVIR **(C)**, and LVEIO **(D)** before and after treatment with Pimobendan. Horizontal lines represent the median; boxes indicate the 25th–75th percentiles; whiskers extend to the most extreme values within 1.5 × the interquartile range. Connecting lines represent changes in individual dogs, with blue points indicating pre-treatment values and red points indicating post-treatment values. The reported *p* value refers to the overall significance assessed using the Wilcoxon signed-rank test. LA/Ao: Left atrial to aortic ratio; LVEIO: Left ventricular inflow-outflow index; LVIDDn: Normalized left ventricular internal diameter in diastole; MAVIR: Mitral-to-aortic velocity-time integral ratio.

Data distribution was assessed graphically and analytically using the Shapiro–Wilk test, and results were presented as mean ± standard deviation or as median (25th–75th percentiles), depending on the distribution. Descriptive statistics were calculated for sex, age, body weight, and echocardiographic parameters before and after treatment. Inferential analysis was conducted within groups before and after treatment to assess differences in echocardiographic parameters using the nonparametric paired Wilcoxon signed-rank test. Boxplots were generated to visually assess data distribution and changes before and after treatment (Figure 1). Baseline clinical and echocardiographic variables were compared between dogs showing reverse remodeling, with echocardiographic indices returning to values within the range consistent with ACVIM stage B1, and those remaining within the stage B2 range after treatment using the Mann–Whitney U test.

Intra-rater reliability of selected echocardiographic indexes was evaluated across ten examinations using the intraclass correlation coefficient (ICC). Results are reported as ICC values with 95% confidence intervals (95% CI). As each examination was measured twice by the same observer, a two-way mixed-effects, single-measurement, absolute-agreement ICC model was used. ICC values were classified as poor (< 0.5), moderate (0.50–0.75), good (0.76–0.90), and excellent (> 0.90).

A *p*-value < 0.05 was considered statistically significant.

## Results

### Population

Between January 2022 and December 2025, 556 dogs with MMVD at ACVIM stage B2 were examined; 42 dogs fulfilled the inclusion criteria. The main reasons for exclusion were ongoing treatment with pimobendan (351), owner refusal or inability to return for the 1-week re-evaluation (56), administration of other concurrent cardiovascular therapies (43), excessive patient stress or poor cooperation preventing repeated echocardiographic examination (33), presence of clinically relevant arrhythmias or marked respiratory sinus arrhythmia (22), presence of other congenital or acquired cardiac diseases (4), significant concomitant valvular regurgitation (3), incorrect pimobendan administration during the first week of treatment (2). Twenty-three were males, and 19 were females. The median age was 145 months (range 124–159); the median weight was 8.5 kg (range 5.82–10.8 kg). Several breeds were represented, including 11 mongrel, 7 Cavalier King Charles spaniel, 6 Chihuahua, 3 Dachshund, 2 Maltese, 2 Pinscher, 2 Shih-Tzu, and 1 of each breed: Cocker spaniel, Schnauzer, Labradoodle, Golden Retriever, Jack Russell terrier, Chinese Crested dog, Epagneul Breton, Poodle, Whippet. The median pimobendan dosage was 0.29 mg/kg twice daily (range 0.25–0.32 mg/kg).

### Echocardiographic indices

Echocardiographic index values before and after treatment are shown in Table 1.

**Table 1:** Echocardiographic indices at the inclusion without any treatment (pre) and after 7 days of therapy with pimobendan (post).

Variable	N	Pre	Post	Wilcoxon *p*-value
LADn (cm/Kg^309^)	42	1.87 [1.64–2.04]	1.72 [1.6–1.97]	<0.001
LA/Ao	42	1.8 [1.67–1.91]	1.55 [1.46–1.68]	<0.001
LVIDDn (cm/Kg^294^)	42	1.86 [1.76–2.02]	1.79 [1.69–2.02]	<0.001
LVIDSn (cm/Kg^315^)	42	1 [0.91–1.12]	0.95 [0.81–1.02]	<0.001
VTI_PA_ (cm)	42	8.39 [6.64–9.31]	9.68 [7.33–10.7]	<0.001
VTI_Ao_ (cm)	42	10.5 [9.47–13.88]	12 [9.62–13.67]	0.267
E_vel_ (m/s)	42	1 [0.85–1.21]	0.96 [0.82–1.09]	0.001
VTI_Mit_	42	13.3 [11.95–15.18]	13.05 [11.48–14.07]	0.246
S′ (cm/s)	42	10.2 [8.92–12.07]	11.71 [9.99–14.05]	<0.001
E/E′	42	9.55 [8.04–11.42]	9.53 [7.42–10.86]	0.031
MPI	42	0.68 [0.57–0.84]	0.71 [0.63–0.88]	0.082
RF (%)	42	37.72 [17.56–56.78]	36.21 [5.77–51.22]	0.196
RV (mL/Kg)	42	0.87 [0.36–1.5]	0.87 [0.1–1.28]	0.175
LAV_max_ (mL/Kg)	42	2.42 [1.68–3.27]	1.9 [1.35–2.96]	<0.001
LAV_min_ (mL/Kg)	42	0.82 [0.56–1.33]	0.65 [0.38–1.12]	0.008
LA-SV (mL/Kg)	42	1.48 [1.04–2.01]	1.3 [0.73–1.73]	0.011
MAVIR	42	1.19 [1–1.52]	1.1 [0.94–1.4]	0.045
LVEIO	42	9.56 [6.77–13.76]	8.16 [5.91–11.11]	<0.001
FS (%)	42	44.9 [38.13–49.05]	47.25 [43.31–51.45]	0.007
EF (%)	42	76.95 [67.56–79.91]	73.55 [68.01–82.4]	0.638
IVRT (msec)	42	54.5 [41.25–63.38]	55 [47–61]	0.899
E/IVRT	42	51.99 [39.17–73.95]	58.7 [42.61–71.66]	0.339
E_dec_ (msec)	42	99.5 [76–117.5]	104 [95.32–123.25]	0.076

Data are presented as median, and interquartile range in brackets. The last column shows the level of significance. Edec, Early diastolic transmitral flow deceleration; E/E’, Early diastolic transmitral flow peak velocity-to-early diastolic myocardial peak velocity ratio; EF, Ejection fraction; E/IVRT, E-to-isovolumic relaxation time ratio; Evel, Peak velocity of early diastolic transmitral flow; FS, Fractional shortening; IVRT, Isovolumic relaxation time; LA/Ao, Left atrial to aortic ratio; LADn, Normalized left atrial diameter; LA-SV, Left atrial stroke volume; LAVmax, Maximal left atrial volume; LAVmin, Minimal left atrial volume; LVEIO, Left ventricular inflow-outflow index; LVIDDn, Normalized left ventricular internal diameter in diastole; LVIDSn, Normalized left ventricular internal diameter in systole; MAVIR, Mitral-to-aortic flow velocity time integral ratio; MPI, Myocardial performance index; RF, Regurgitant fraction; RV, Regurgitant volume; S′, Systolic myocardial peak velocity; VTIAo, Velocity-time integral of aortic flow; VTIMit, Velocity-time integral of mitral inflow; VTIPA, Velocity-time integral of pulmonic flow.

At the 1-week recheck, a reduction of LA and LV size was observed with a reduction in LADn (*p* < 0.001), LA/Ao (*p* < 0.001), LAV_max_ (p < 0.001), LAV_min_ (*p* = 0.008), LA-SV (*p* = 0.011), LVIDDn (*p* < 0.001), and LVIDSn (p < 0.001). Additionally, there was a significant increase in FS% (*p* = 0.007), E_Vel_ (*p* = 0.001), E/E′ (*p* = 0.031), LVEIO (p < 0.001), and MAVIR (*p* = 0.045). The VTI_PA_ (*p* < 0.001), and S′ (*p* < 0.001) increased. Conversely, there was no difference for VTI_Ao_, VTI_Mit_, MPI, IVRT, E/IVRT, E_dec_, EF%, RV, and RF. From the first echocardiographic exam and 1-week recheck, 22 dogs (52.4%) have returned to echocardiographic indices within the range consistent with stage B1.

The results of the subgroup analysis comparing dogs with reverse remodeling and those without were reported in Table 2. Dogs showing reverse remodeling had significantly lower baseline LA/Ao (*p* = 0.020), LAV_max_ (*p* = 0.045), LAV_min_ (*p* = 0.010), and E_dec_ (*p* = 0.027), while no significant differences were observed for the remaining baseline variables.

**Table 2:** Baseline comparison between dogs that showed reverse remodeling, with echocardiographic indices that returned within the range consistent with stage B1, and dogs without reverse remodeling.

Baseline variable	Reverse remodeling (22)	No reverse remodeling (20)	*P* value
Age (months)	147.5 [132.2–158.2]	135.5 [100.5–160.8]	0.279
Weight (Kg)	8.2 [5.7–10.2]	8.8 [6.6–11.9]	0.302
Drug dose (mg/Kg)	0.3 [0.2–0.3]	0.3 [0.3–0.3]	0.575
LADn (cm/Kg^309^)	1.8 [1.6–2.0]	1.9 [1.8–2.1]	0.083
LA/Ao	1.7 [1.6–1.9]	1.8 [1.8–2.1]	0.020
LVIDDn (cm/Kg^294^)	1.9 [1.7–1.9]	2.0 [1.8–2.0]	0.095
LVIDSn (cm/Kg^315^)	1.0 [0.9–1.1]	1.1 [0.9–1.1]	0.286
VTI_PA_ (cm)	8.7 [7.1–10.0]	7.7 [6.4–8.8]	0.284
VTI_Ao_ (cm)	10.5 [9.3–14.1]	10.4 [9.8–13.5]	0.840
E_vel_ (m/s)	1.0 [0.8–1.1]	1.1 [0.9–1.2]	0.207
VTI_Mit_ (cm)	12.8 [11.5–15.5]	14.1 [12.4–14.8]	0.529
S′ (cm/s)	10.0 [8.5–12.0]	10.3 [9.7–12.3]	0.284
E/E′	9.6 [7.4–12.2]	9.4 [8.5–10.9]	0.980
MPI	0.7 [0.6–0.8]	0.7 [0.6–0.8]	0.758
RF (%)	34.5 [16.2–56.1]	39.2 [20.5–56.8]	0.811
RV (mL/Kg)	0.8 [0.3–1.2]	0.9 [0.5–1.7]	0.457
LAV_max_ (mL/Kg)	2.3 [1.6–2.7]	2.8 [2.0–3.8]	0.045
LAV_min_ (mL/Kg)	0.6 [0.5–1.0]	1.1 [0.7–2.0]	0.010
LA-SV (mL/Kg)	1.5 [1.0–1.9]	1.4 [1.1–2.1]	0.791
MAVIR	1.2 [1.1–1.6]	1.1 [1.0–1.4]	0.733
LVEIO	9.2 [6.3–13.8]	9.9 [7.1–12.4]	0.687
FS (%)	44.2 [39.4–48.7]	45.9 [36.5–50.6]	0.990
EF (%)	75.7 [69.0–79.7]	77.8 [67.4–80.3]	0.840
IVRT (msec)	54.0 [42.0–63.0]	56.0 [40.0–64.4]	0.910
E/IVRT	52.0 [41.1–75.0]	51.1 [38.7–70.7]	0.641
E_dec_ (msec)	111.0 [86.8–138.5]	88.5 [74.0–102.2]	0.027

Data are presented as median, and interquartile range in brackets. The last column shows the level of significance. Edec, early diastolic transmitral flow deceleration; E/E’, early diastolic transmitral flow peak velocity-to-early diastolic myocardial peak velocity ratio; EF, ejection fraction; E/IVRT, E-to-isovolumic relaxation time ratio; Evel, peak velocity of early diastolic transmitral flow; FS, fractional shortening; IVRT, isovolumic relaxation time; LA/Ao, left atrial to aortic ratio; LADn, normalized left atrial diameter; LA-SV, left atrial stroke volume; LAVmax, maximal left atrial volume; LAVmin, minimal left atrial volume; LVEIO, left ventricular inflow-outflow index; LVIDDn, normalized left ventricular internal diameter in diastole; LVIDSn, normalized left ventricular internal diameter in systole; MAVIR, mitral-to-aortic flow velocity time integral ratio; MPI, myocardial performance index; RF, regurgitant fraction; RV, regurgitant volume; S′, systolic myocardial peak velocity; VTIAo, velocity-time integral of aortic flow; VTIMit, velocity-time integral of mitral inflow; VTIPA, velocity-time integral of pulmonic flow.

Intra-observer reliability was excellent for all variables, with an ICC > 0.9, except for LA/Ao, for which reliability was moderate (ICC = 0.692) (Table 3).

**Table 3:** Intra-observer reliability of selected echocardiographic indices in ten dogs with subclinical myxomatous mitral valve disease.

Variable	N	ICC (95% CI)
LAD	10	0.989 (0.958–0.997)
Annulus	10	0.985 (0.945–0.996)
LVIDD	10	0.996 (0.982–0.999)
LVIDS	10	0.989 (0.958–0.997)
LA/Ao	10	0.692 (0.199–0.912)
TSV	10	0.979 (0.923–0.995)
LAVmax	10	0.932 (0.709–0.983)
LAVmin	10	0.983 (0.938–0.996)

ICC, Intraclass correlation coefficient; LAD, Left atrial diameter; LA/Ao, Left atrial to aortic root ratio; LAVmax, Maximal left atrial volume; LAVmin, Minimal left atrial volume; LVIDD, Left ventricular internal diameter in diastole; LVIDS, Left ventricular internal diameter in systole; TSV, Total stroke volume.

## Discussion

This prospective observational study evaluated the short-term echocardiographic effects of pimobendan administration in dogs with ACVIM stage B2 MMVD.

The main finding of this study is that pimobendan administration was associated with a measurable improvement in several echocardiographic indices of cardiac remodeling after 7 days of treatment. In particular, LA and LV size showed a significant reduction, suggesting an early effect of the drug on cardiac loading conditions and confirming the findings reported in previous studies (3, 5).

In dogs with MMVD, chronic volume overload and increased preload contribute to disease progression and the development of CHF. Pimobendan is an inodilator with a documented effect on reducing the regurgitant fraction (7). Experimental studies in dogs with induced mitral regurgitation have proposed that reduced heart size and enhanced contractile function are associated with a reduction in mitral regurgitant orifice area, ultimately resulting in decreased regurgitant volume (15, 16). Left heart enlargement is a hallmark of MMVD progression and reflects chronic volume overload due to mitral regurgitation (17). In the present study, several parameters of LA size, including LA/Ao, LADn, and LA volumes, as well as LV dimensions, were significantly reduced at follow-up, supporting the hypothesis that pimobendan may reduce volume overload and promote reverse remodeling through a decrease in mitral regurgitation severity. This effect could be hypothesized to be the main determinant of the beneficial effects on disease progression and survival time.

Notably, considering the parameters used to classify the disease, the median LA/Ao ratio and LVIDDn decreased below the diagnostic cut-off for stage B2, and 22 dogs have returned to echocardiographic indices within the range consistent with stage B1. A previous study suggested that those animals with a documented reduction in the left heart chambers after pimobendan treatment, who no longer met the criteria for stage B2 disease, should instead be described as “stage B2 reverse remodeled” (5). Stage B2 includes dogs with varying degrees of left heart enlargement, ranging from mild to severe, and this population heterogeneity may have influenced the percentage of dogs showing reverse remodeling. The subgroup analysis showed that dogs with reverse remodeling had significantly lower baseline LA/Ao, LAV_max_ and LAV_min_ values than dogs without reverse remodeling, suggesting that less advanced cardiac remodeling at diagnosis may increase the likelihood of an early echocardiographic response to pimobendan. While baseline disease severity may therefore have influenced the proportion of dogs showing reverse remodeling, these findings support the hypothesis that pimobendan can reduce volume overload in the short term and induce reverse remodeling in a proportion of patients.

However, one previous study failed to detect significant changes in left atrial size or function over 6 months in dogs with preclinical MMVD treated with pimobendan, despite reductions in left ventricular dimensions and improvements in systolic function. Nevertheless, the absence of negative changes may still indicate a beneficial role of pimobendan in slowing disease progression (6).

The degree of cardiac dilation is mainly influenced by the severity of mitral regurgitation. Although changes in quantitative volumetric measurements of mitral regurgitation severity, such as regurgitant volume and regurgitant fraction, did not reach statistical significance, a marked reduction in left atrial and ventricular volumes was observed. In contrast, MAVIR and LVEIO showed a significant decrease, supporting a reduction in regurgitant severity and cardiac remodeling. MAVIR was proposed in humans as a surrogate for the regurgitant fraction (18–20) and has recently been shown to correlate with echocardiographic indices of volume overload. It also has strong predictive ability in discriminating mitral regurgitation severity in dogs (13). Similarly, the LVEIO, previously used in humans as an indicator of mitral regurgitation severity, was recently found to strongly correlate with regurgitant fraction in dogs (21, 22).

In the present study, volumetric quantitation of mitral regurgitation failed to demonstrate a reduction in RF after 7 days of pimobendan treatment, despite a decrease observed in the MAVIR and LVEIO RF surrogates support this hypothesis. This apparent discrepancy may reflect the complexity of volumetric quantification of mitral regurgitation, the methodological limitations associated with these measurements (23), and the relatively small sample size of the study. Several explanations may account for this finding. First, calculation errors may have been amplified by the use of different methods to derive total and forward stroke volume, including the Simpson method and Doppler-derived measurements at the aortic annulus. Because regurgitant volume was calculated from the difference between total and forward stroke volume, relatively small concomitant changes in both these variables may have limited the ability to detect significant differences. Furthermore, the regurgitant fraction incorporates the total stroke volume in both the numerator and denominator, potentially preserving the calculated value despite reductions in ventricular and atrial volumes. Second, multiple hemodynamic effects of pimobendan may influence regurgitant indices in opposite directions: a reduction in preload may decrease ventricular filling and total stroke volume through the Frank–Starling mechanism, whereas its inodilator effects may reduce afterload and improve systolic performance, thereby preserving forward stroke volume. In the present study, the greater reduction in end-systolic dimensions compared with end-diastolic dimensions may suggest improved systolic emptying. However, because end-diastolic volume also decreased, the total ejected volume may have remained relatively unchanged despite the apparent improvement in systolic performance. Although regurgitant volume and regurgitant fraction did not change significantly after treatment, VTI_PA_ increased significantly, and VTI_Ao_ showed a mild numerical increase, suggesting improved forward hemodynamics after pimobendan administration. Therefore, a modest reduction in regurgitant volume and regurgitant fraction cannot be excluded. The smaller increase in VTI_Ao_ may reflect the greater influence of mitral regurgitation and left ventricular loading conditions on this parameter, whereas VTI_PA_ may represent a more stable marker of forward flow. However, because the pulmonary and systemic circulations are connected in series, right and left ventricular forward stroke volumes are ultimately expected to be similar. Consequently, the study may not have had sufficient sensitivity to detect small but clinically relevant short-term changes in regurgitant indices.

Another aspect suggesting improved systolic performance in the present study was the increase in both FS% and S′ after treatment, indicating improvement in both radial/circumferential and longitudinal function. These parameters tend to increase with the progression of MMVD, reflecting a compensatory hyperdynamic state associated with chronic volume overload due to mitral regurgitation (24–26). In our study, the observed increase in these indices after a week of treatment may reflect the pharmacological effects of pimobendan; however, these findings should be interpreted in conjunction with other echocardiographic parameters to provide a more comprehensive assessment of systolic function. Both FS% and S′ are influenced by loading conditions, and their increases may reflect the combined effects of enhanced contractility and reduced afterload associated with pimobendan administration, as well as the hemodynamic effects of mitral regurgitation itself. In contrast, ejection fraction did not show a similar increase and, being derived from volumetric measurements, may have been more susceptible to preload dependence and methodological variability.

E-wave transmitral peak velocity has been demonstrated to be an important prognostic factor in dogs with MMVD, with values exceeding 1.2 m/s reported to indicate a poorer prognosis, and increasing mitral E_Vel_ values over serial examinations associated with a poorer outcome (27, 28). E-wave transmitral peak velocity is determined mainly by LV filling pressures and relaxation. In MMVD, where the preload is increased, the filling pressure component is dominant on relaxation, and an increase in E_Vel_ is an indirect measure of mitral regurgitation severity, reflecting worsening pressure differences between the LA and LV with increasing regurgitant volumes (29). Peak E-wave velocity significantly decreased after treatment in our population, suggesting a reduction in LV filling pressures and volume overload. However, the E/E′ ratio did not change significantly, indicating that not all indices of diastolic function responded uniformly over the short observational period. In particular, this study was conducted on a population of stage B2 dogs at the first diagnosis, in which we can assume that the diastolic function was preserved. Unlike E velocity, E′ and IVRT are considered less load-dependent and more reflective of intrinsic diastolic function. Therefore, the reduction in only E/E′, without a corresponding decrease in E/IVRT, may be more consistent with changes in loading conditions than with a true improvement in diastolic function (25, 30). However, a direct lusitropic effect of pimobendan cannot be conclusively demonstrated from these findings, particularly in the absence of a direct measure of relaxation such as *τ* (tau).

This study has several limitations. First, the absence of a placebo-controlled group limits the ability to attribute the observed echocardiographic changes exclusively to pimobendan treatment. However, the inclusion of a control group was considered ethically challenging, as current clinical recommendations support pimobendan administration in dogs with ACVIM stage B2 MMVD. Second, the number of included dogs was limited because many dogs were excluded for different reasons, despite the larger population of stage B2 dogs initially evaluated. Third, the interpretation of regurgitant indices was based on volumetric calculations derived from two-dimensional echocardiography rather than cardiac magnetic resonance imaging, which is considered the reference standard for volumetric assessment of regurgitation (31–33). Finally, the heterogeneity of the stage B2 population included dogs with varying degrees of cardiac remodeling, including several dogs with only mild left heart enlargement, which may have influenced the ability of regurgitant fraction and regurgitant volume to detect treatment-related changes. This could also be the reason why, in this study, such a high proportion of subjects had echocardiographic indices falling within the range consistent with stage B1 after 1 week of treatment.

In conclusion, short-term pimobendan administration was associated with left atrial and ventricular reverse remodeling, resulting in 52% of the dogs showing echocardiographic parameters that returned to values consistent with MMVD stage B1. The reduction in indices associated with mitral regurgitation severity, such as MAVIR and LVEIO, may be consistent with an underlying reduction in regurgitant volume that was not directly detected by regurgitant fraction measurements.

## Data Availability

The raw data supporting the conclusions of this article will be made available by the authors, without undue reservation.
